# Applying a 4D multiscale *in vivo* tumor growth model to the exploration of radiotherapy scheduling: The effects of weekend treatment gaps and p53 gene status on the response of fast growing solid tumors

**Published:** 2007-02-16

**Authors:** Dimitra D. Dionysiou, Georgios S. Stamatakos

**Affiliations:** 1 *In Silico* Oncology Group, Laboratory of Microwaves and Fiber Optics, Institute of Communication and Computer Systems, School of Electrical and Computer Engineering, National Technical University of Athens, GR-157 80 Zografos, Greece; 2 Department of Mathematics, School of Applied Sciences, National Technical University of Athens, GR-157 80 Zografos, Greece

**Keywords:** simulation, radiotherapy, p53, fractionation, HART, CHART, glioblastoma

## Abstract

The present paper aims at demonstrating clinically oriented applications of the multiscale four dimensional *in vivo* tumor growth simulation model previously developed by our research group. To this end the effect of weekend radiotherapy treatment gaps and p53 gene status on two virtual glioblastoma tumors differing only in p53 gene status is investigated *in silico.* Tumor response predictions concerning two rather extreme dose fractionation schedules (daily dose of 4.5 Gy administered in 3 equal fractions) namely HART (Hyperfractionated Accelerated Radiotherapy weekend less) 54 Gy and CHART (Continuous HART) 54 Gy are presented and compared. The model predictions suggest that, for the same p53 status, HART 54 Gy and CHART 54 Gy have almost the same long term effects on locoregional tumor control. However, no data have been located in the literature concerning a comparison of HART and CHART radiotherapy schedules for glioblastoma. As non small cell lung carcinoma (NSCLC) may also be a fast growing and radiosensitive tumor, a comparison of the model predictions with the outcome of clinical studies concerning the response of NSCLC to HART 54 Gy and CHART 54 Gy is made. The model predictions are in accordance with corresponding clinical observations, thus strengthening the potential of the model.

## Introduction

The importance of efficient modeling of biological phenomena related to tumor response to radiotherapy is nowadays widely accepted. During the past four decades researchers have enhanced the understanding of tumor growth as well as tumor response to radiation therapy by means of various simulation models. Representative examples drawn from the extensive corresponding literature have been given in (Stamatakos et al. 2002, Dionysiou et al. 2004). The need for novel multi-disciplinary computational models simulating tumor growth and response to therapy has been stressed by many researchers.

The aim of the present paper is to present comparative results of a recently developed model *of in vivo* tumor growth and response to irradiation. Emphasis is placed on weekend treatment gaps in conjunction with p53 gene status. The model is based on the available imaging, histopathologic and genetic data of the patient and numerous fundamental biological mechanisms are incorporated and explicitly described. The long-term goal of this work is twofold: the development of a computer tool for getting insight into cancer biology and of an advanced patient-specific decision support system.

## A brief outline of the *in silico* model

In the following paragraphs a brief outline of the *in silico* model is presented through the consideration of the glioblastoma (GB) paradigm. Two of the main reasons for the GB consideration have been the availability of adequate imaging data and the existence of reliable molecular-radiobiological data for two glioblastoma lines differing only in p53 status.

In the general case, the available imaging, histopathologic and genetic data of the patient are appropriately collected. The clinician delineates the tumor and its metabolic subregions by using a dedicated computer tool. In the case of radiotherapy, the distribution of the absorbed dose in the region of interest is also acquired. Random number generators are used in order to simulate the statistical nature of various phenomena. For a detailed description refer to (Stamatakos et al. 2002; Dionysiou et al. 2004). For the purpose of the 3D reconstruction and visualization the 3D visualization package AVS/Express is used (Dionysiou et al. 2003).

A three-dimensional discretizing mesh covers the region of interest. The elementary cubic volume of the mesh is called “Geometrical Cell (GC)”. During the simulation procedure the geometrical mesh is scanned every T units of time. In each time step, the updated state of a given GC is determined on the basis of a number of algorithms describing the behavior of the cells constituting the tumor, which are briefly presented in the following paragraphs. Each GC of the mesh initially accommodates a Number of Biological Cells (NBC). NBC apparently depends on the chosen size of the GC and determines the quantization error of the model. Each GC of the mesh belonging to the tumor contains biological cells, which are distributed in a number of “classes” (compartments), each one characterized by the phase in which its cells are found (within or out of the cell cycle: G_1_, S, G_2_, M, G_0_, Necrosis, Apoptosis).

**Figure 1.**
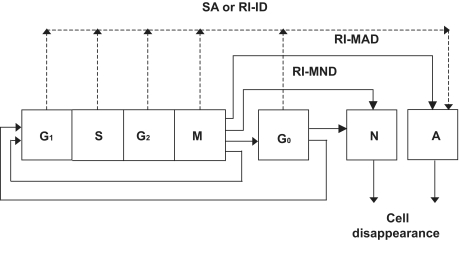
Cytokinetic model of a tumour cell. G_1_: G_1_ (gap1) phase, S: DNA synthesis phase, G_2_: G_2_ (gap 2) phase, M (mitosis), G_0_: resting phase, N: necrosis, SA: spontaneous apoptosis, RI-ID: radiation-induced interphase death, RI-MAD: radiation-induced mitotic apoptotic death, RI-MND: radiation-induced mitotic necrotic death.

The cytokinetic model of Figure 1, originally introduced in (Dionysiou et al. 2005), is used. Proliferating tumor cells pass through the phases G_1_ (gap 1), S (DNA synthesis), G_2_ (gap 2), and M(mitosis). After mitosis, each one of the daughter cells re-enters G_1_ if the oxygen and nutrient supply in its position is adequate. Otherwise, it enters the resting G_0_ phase, where it can stay for a limited time, T_G0_; it then enters the necrotic phase leading to cell lysis, unless in the meantime the local environment has become favorable. In the latter case, the cell re-enters G_1._ Two basic mechanisms of radiation-induced cell death are being treated: apoptotic and necrotic cell death. Apoptotic cell death is subdivided into radiation-induced inter-phase death (RI-ID) (direct death through apoptosis) and radiation-induced mitotic apoptotic death (RI-MAD) (Dewey 1995, Steel 2001). In most solid tumors the majority of lethally damaged cells dies through a radiation-induced mitotic necrotic mechanism (RI-MND) and is considered to undergo a few mitotic divisions prior to death and disappearance from the tumor. In the present model these cells are assumed to complete two mitotic divisions before dying. The assumption of two mitotic divisions as a typical division number before the death of lethally injured irradiated cells is based on relevant data derived from the literature (Denekamp 1986, Perez and Brady, 1998, p.87), which states that cells irradiated with low radiation doses (e.g. 1–10 Gy) may successfully complete one or two divisions before death, whereas after high doses they die at the first attempted division. The fraction dose of 1.5 Gy used in HART and CHART radiotherapy schedules is at the lower limit of the above-mentioned low-dose interval (1–10 Gy), so the assumption of two mitotic divisions instead of one seems more biologically relevant.

Cell loss from the tumor due to apoptosis and necrosis is estimated based on the the cell loss factor due to necrosis (CLFN) and apoptosis (CLFA) according to [Steel 2002, p. 13]. Specifically, the probability of cell loss per hour due to necrosis/apoptosis represents the cell loss rate due to necrosis/apoptosis and is the product of the cell loss factor due to necrosis/apoptosis and the cell birth rate. The cell birth rate (CBR) can be considered as the ratio of the growth fraction (GF) to the cell cycle duration (TC), i.e.: *CLF* = *CLR/CBR, CBR* = *GF/T_C_*.

The distribution of the initial NBC cells of a GC in each phase class is estimated according to the position of the corresponding GC, namely based on the estimated local metabolic activity (e.g. through PET or SPECT or indirectly through the use of contrast enhanced T1 weighted MRI). Furthermore, the initial distribution of the proliferating cells within each one of the proliferating phases (G_1_, S, G_2_, M) is estimated using the mean duration of each cell cycle phase for the specific tumor.

Cell killing by irradiation is described by the Linear Quadratic or LQ Model (Steel 2002):

(1) $$S(D)=\text{exp}[-(\alpha D+\beta D^{2})]$$

where S(D) is the surviving fraction after a (uniform) dose D (Gy) of radiation to a population of cells. The parameters α (Gy-1) and β (Gy-2) are called the radiosensitivity parameters of the LQ model. Cell radiosensitivity varies considerably throughout the cell cycle. The S phase is regarded as the most resistant. Cells in any proliferating phase are more radiosensitive than hypoxic cells residing in G_0_ (Steel 2002). Based on these observations we have used different values for the radiosensitivity parameters of the LQ model for the S phase (*α_S_*, *β_S_*), the proliferating phases G_1_, G_2_, M (*α_P_*, *β_P_*), and the G_0_ phase (*α_G0_*, *β_G0_*). Specifically, the values *of α_S_*, *β_S_* and *α_G0_*, *β_G0_* have been derived as perturbations of the (*α_P_* *, β_P_*) values.

The basis of the tumor expansion-shrinkage algorithms is described below: In case that the actual number of alive and dead tumor cells contained within a given GC is reduced to less than *NBC/10*, then a procedure which attempts to “unload” the remaining biological cells in the neighboring GCs takes place. The basic criterion of the unloading procedure is the available free space within the neighboring GCs, so that the biological cell density is approximately uniform throughout the geometrical mesh. Therefore, cells are preferentially placed within the neighboring GCs with the maximum available free space. In case that at the end of the unloading procedure the given GC becomes empty, it is assumed to disappear from the tumor. An appropriate shift of a chain of GCs, intended to fill the “vacuum”, leads to tumor shrinkage. This can happen after the killing of a number of cells by irradiation.

On the other hand, if the number of alive and dead cells within a given GC exceeds *NBC + NBC/10*, then a similar procedure attempting to unload the excess cells in the surrounding GCs takes place. In case that the unloading procedure fails to reduce the number of cells to less than *NBC + NBC/10*, then a new GC emerges. Its position relative to the “mother” GC is determined using a random number generator. An appropriate shifting of a chain of adjacent GCs leads to a differential expansion of the tumor. The “newborn” GC initially contains the excess number of biological cells, which are distributed in the various phase classes proportionally to the distribution in the “mother” GC. The procedure for choosing the appropriate shifting direction and the reason for choosing the cutoff values *NBC/10* and *NBC + NBC/*10 are analytically presented in (Dionysiou et al. 2005).

## Simulation Executions

### p53 gene status and radiosensitivity

The role of oncogenes and tumor suppressor genes in modulating GB radiosensitivity constitutes the subject of intense research efforts. Perhaps the best-studied tumor-suppressor gene in the case of GB is p53. The roles of wild-type (wt) p53 in modulating DNA repair, apoptosis, and the G_1_ cell cycle arrest have each been implicated in the regulation of cellular response to ionizing radiation. An abnormal p53 has been related to a wide variety of tumors throughout the body. p53 mutations in human malignancies are frequently being associated with poor prognosis, poor response to therapy and advanced stage of disease (D’Avenia et al. 2006).

The parameters α and β of the LQ Model constitute one possible way to incorporate the influence of genetic determinants, such as the p53 gene status, into the simulation model. As we have already described (Dionysiou et al. 2004, Dionysiou et al. 2005), a remarkable number of studies associate p53 mutations with increased radioresistance and poor clinical outcome for patients with GB. (Haas-Kogan et al. 1996) observed an increased radioresistance in irradiated GB G_1_ cells lacking functional wild type (wt) p53, manifested by a relatively lower a and α/β. More precisely this observation refers only to two isogenic glioblastoma cell lines (U87-LUX.8 demonstrating wt p53 function and U87-175.4 lacking wt p53 function henceforth denoted by “mt p53”) differing only in p53 status. The following analysis refers to those two genetic profiles. Furthermore, in (Haas-Kogan et al. 1999) they studied the influence of p53 function on the effect of fractionated radiotherapy of GB tumors and concluded that fractionated radiotherapy provides a selective advantage to GB cells expressing mutant p53 (mt p53).

Based on these studies, we considered two hypothetical GB tumors with different p53 status, in order to do a comparative study of the effect of weekend treatment gaps in their response to radiotherapy:

- a GB tumor with wild type p53 (Haas-Kogan et al. 1996 and perturbations):
  *α_p_* = 0.61Gy^−1^, *α_S_* = 0.4472Gy^−1^, *α_G0_* = 0.203Gy^−1^
  *β_p_* = 0.02Gy^−2^, *β_S_* = 0.0128Gy^−2^, *β_G0_* = 0.002Gy^−2^
- a GB tumor with mutant p53 (Haas-Kogan et al. 1996 and perturbations):
  *α_p_* = 0.17Gy^−1^, *α_S_* = 0.1248Gy^−1^, *α_G0_* = 0.057Gy^−1^
  *β_p_* = 0.022Gy^−2^, *β_S_* = 0.0128Gy^−2^, *β_G0_* = 0.002Gy^−2^

where: α*_p_*, β*_p_*: the LQ Model parameters for G_1_*_,_*G_2_, M phases, α*_S_*, β*_S_*: the LQ Model parameters for S phase, α*_G0_*, β*_G0_*: the LQ Model parameters for G_0_ phase.

In consistence with experimental biology, we assumed α*_G0_* = α*_p_/OER* and β*_G0_* = β*_p_/OER*^2^, where *OER*: the Oxygen Enhancement Ratio, taken equal to 3 (Perez and Brandy 1998; Kocher et al. 2000; Steel 2002), and α*_S_* = 0.6α*_p_*+ 0.4α*_G0_*, β*_S_* = 0.6 β*_p_* + 0.4 β*_G0_*.

### Other model parameters

A 3D mesh quantizing the anatomical region of interest has been considered. The dimensions of each GC are 1mm × 1mm × 1mm. Such a volume contains roughly 10^6^ biological cells (*NBC =* 10^6^) (Steel 2002). Since GB is generally considered a poorly differentiated tumor (Curran R.C. and Crocker J. 2000), as a first approximation all non-clonogenic cells are considered to be necrotic (sterile cells are not taken into account). A typical clonogenic cell density is 10^4^ cells/mm^3^ (Jones and Dale 1999). Since GB tumors are highly aggressive and rapidly growing, we assume a clonogenic cell density of 2 × 10^5^ cells/mm^3^ in the proliferating cell region (a 6 mm thick layer from the outer boundary of the tumor towards its interior), 10^5^ cells/mm^3^ in the G0 cell region (a 1 mm thick layer surrounding the central necrotic region) and 0.2 × 10^5^ cells/mm^3^ in the dead cell region of the tumor. In the proliferating cell region, 70% of the clonogenic cells are assumed to be in the cycling phases and 30% in the G_0_ phase. In the G_0_ cell region, 30% of the clonogenic cells are in the cycling phases and 70% in the G_0_ phase. Finally, in the dead cell region, 10% of the clonogenic cells are in the cycling phases and 90% in the G_0_ phase. The cell cycle duration, T_C_, is taken equal to 40h. The approximate percentage of the cell cycle time spent in each phase by a typical malignant cell can be given by: *T_G1_* = 0.4*T_C_*, *T_S_* = 0.39*T_C_*, *T_G2_* = 0.19*T_C_*, *T_M_* = 0.02*T_C_* (Salmon and Sartorelli 2001). The duration of the G_0_ phase is taken to be *T_G0_* = 25h (Duechting et al. 1994).

The total cell loss factor (CLF), the sum of the cell loss factor due to necrosis and the cell loss factor due to apoptosis, has been taken equal to 0.3 at the simulations presented, as this is a reasonable value for glioblastoma multiforme tumors [Huang et al. 1995]. Futhermore, in glioblastoma tumors treated by irradiation there generally are low levels of apoptotic cells [Yew et al. 1998, Salmon and Sartorelli 2001]. Therefore we have used CLFN = 0.27 and CLFA= 0.03, or in general CLFN = 0.9 CLF, CLFA = 0.1CLF.

The Hyperfractionated Accelerated Radiotherapy (HART) scheme (dose fraction 1.5 Gy, three fractions per day, 6h interval between any two consecutive fractions in the same day, 5 days per week, 54 Gy in total) and the Continuous Hyperfractionated Accelerated Radiotherapy (CHART) scheme (dose fraction 1.5 Gy, three fractions per day, 6h interval between any two consecutive fractions in the same day, 7 days per week, 54 Gy in total) have been simulated. The distribution of the absorbed dose in the tumor region is assumed to be uniform. HART and CHART were considered because they are convenient for the study of the effect of weekend treatment gaps on tumor response to radiotherapy.

The simulation is assumed to begin (t = 0) on Monday 00:00 a.m. and end on Sunday 24:00, 12 weeks later. The computer code has been developed in Microsoft Visual C++ 6 and Visual Basic 6 programming languages. An execution of a radiation therapy simulation of 6 weeks (96 × 96 × 96 GCs, each one of dimensions 1mm × 1mm × 1mm) on an Intel Pentium 4, 2.8GHz HT (512MB RAM) takes about 10min.

### Results

**Figure 2.**
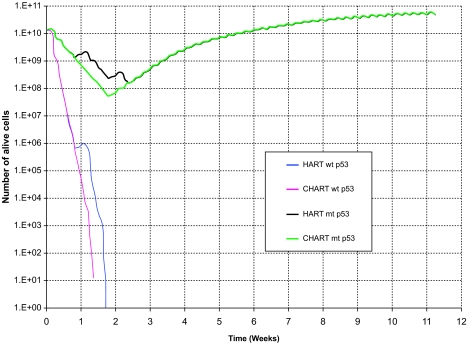
The number of alive tumor cells as a function of time for two hypothetical tumors differing in their p53 status. HART and CHART dose fractionation schedules. The curves corresponding to HART and CHART practically coincide over substantial time intervals for the same p53 gene status.

**Figure 3.**
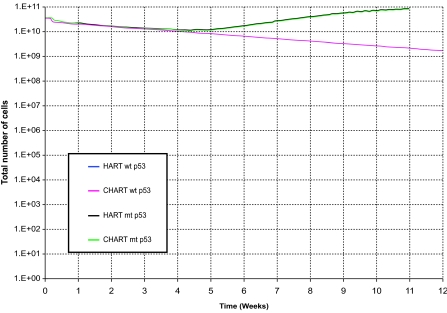
The number of total tumor cells as a function of time for two hypothetical tumors differing in their p53 status. HART and CHART dose fractionation schedules. The curves corresponding to HART and CHART practically coincide for the same p53 gene status.

**Figure 4.**
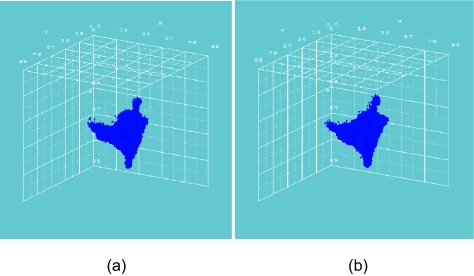
3D reconstruction of the tumor with wt p53, 6 weeks after the beginning of the simulation: (a) HART radiotherapy scheme, (b) CHART radiotherapy scheme. Color code: red: proliferating cell region, green: G_0_ cell region, blue: dead cell region.

**Figure 5.**
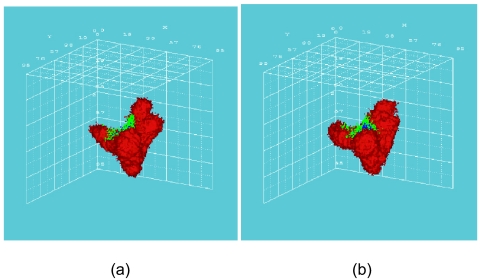
3D reconstruction of the tumor with mt p53, 6 weeks after the beginning of the simulation: (a) HART radiotherapy scheme, (b) CHART radiotherapy scheme. Color code: red: proliferating cell region, green: G_0_ cell region, blue: dead cell region.

Fig. 2 and Fig. 3 depict the number of alive and total tumor cells respectively, as a function of time, for the HART and CHART fractionation schemes, and for the two hypothetical GB tumors differing in their p53 status. In Fig. 4 the 3D reconstruction of the tumor with wt p53 6 weeks after the beginning of the simulation of the HART and CHART schedules is presented. Fig. 5 depicts the same 3D reconstructions for the tumor with mt p53. As expected, 3D visualization offers improved insight into the macroscopic geometry and structure of the tumor.

The results of the comparative simulations, as depicted in the cell number diagrams and the 3D reconstructions of the tumors, are biologically reasonable. In the case of the tumor with wt p53, which is considered to be more radiosensitive compared with the tumor with mt p53, the trend for reduction of the number of living tumor cells is clearly pronounced. In fact, the tumor with wt p53 is so radiosensitive that both HART and CHART fractionation schedules seem to eliminate “all” the clonogenic cells that the tumor initially contained. Obviously the apparent complete elimination of living cells is to be considered more a consequence of the model quantization than a quantitatively exact prediction. On the contrary, the tumor with mt p53 is very radioresistant and as a result the reduction in the number of tumor cells during radiotherapy is modest. After the end of both radiotherapy schedules the surviving tumor cells begin to repopulate the tumor.

As has already been mentioned, no data have been located in the literature concerning a comparison of HART and CHART radiotherapy schedules for glioblastoma. However, as NSCLC may also be a fast growing and considerably radiosensitive tumor, we resorted to a rough comparison of the simulation model predictions with the outcome of clinical studies concerning the response of NSCLC to CHART 54 Gy and CHARTWEL (=HART) 54 Gy (Turrisi 1999). The model predictions suggest that for the same p53 status HART and CHART have almost the same long term effects on locoregional tumor control. This is in accordance with Turrisi’s clinical studies’ observations as well as with common intuition. Obviously well designed experimental work on tumor spheroids developed from the glioblastoma cell lines considered might be a clear path to refine model validation and to provide feedback for optimization. As for the same p53 status HART and CHART seem to produce similar long term effects on locoregional tumor control, the main criterion for selecting one out of the two schemes considered is sparing of the adjacent normal tissues.

The effect of the treatment gap on the number of alive tumor cells is evident. Both schemes employ the same total dose and fraction dose, but in the case of CHART there is no treatment gap during the weekend; the irradiation of the tumor takes place twelve consecutive days. Therefore, CHART seems to be advantageous in terms of tumor cell repopulation restrain during therapy. Nevertheless, since CHART’s duration is shorter, if it does not succeed in eliminating all clonogenic tumor cells – as in the case of tumor with mt p53-repopulation of the tumor begins earlier.

**Figure 6.**
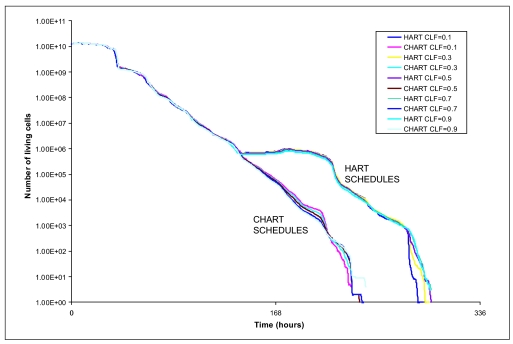
Explorative HART and CHART radiotherapy simulations. Number of living tumor cells as a function of time, various CLF values.

**Figure 7.**
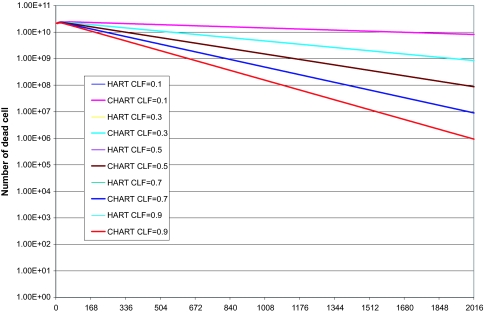
Explorative HART and CHART radiotherapy simulations. Number of dead tumor cells as a function of time, various CLF values.

**Figure 8.**
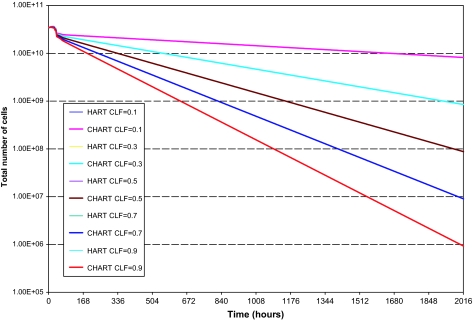
Explorative HART and CHART radiotherapy simulations. Total number of tumor cells as a function of time, various CLF values.

Finally, to investigate the effect of the cell loss factor value on the model’s output we performed a series of explorative HART and CHART radiotherapy simulations presented in the following figures (fig. 6–8).

CLF value mainly influences the tumor shrinkage rate, since it relates to the speed by which dead cells disappear from the tumor. The higher is the value of CLF, the higher the shrinkage rate of the tumor during radiotherapy. CLF’s effect on the number of *living* cells (proliferating and G0) throughout radiotherapy it’s minimal. Therefore its effect on the comparative outcome of the HART and CHART schedules is minimal as well. In the diagrams of the number of dead cells and the total number of cells in the tumor the lines representing HART or CHART schedules with the same CLF value almost coincide.

## Conclusion

The simulation results concerning locoregional tumor control are in agreement with experimental observations and clinical experience. Since no data have been located in the literature concerning a comparison of HART and CHART radiotherapy schedules for glioblastoma multiforme, we resorted to a comparison of the simulation model predictions with the outcome of clinical studies concerning the response of NSCLC to CHART 54 Gy and CHARTWEL (=HART) 54 Gy, guided by the fact that NSCLC is a fast growing and considerably radiosensitive tumor, as glioblastoma generally is considered. The model predictions suggest that, for the same p53 status, HART and CHART have almost the same long term effects on locoregional tumor control. Apart from intuitive, this is also in accordance with clinical observations.

The model satisfactorily simulates characteristics of tumor behavior such as tumor shrinkage, repopulation and expansion and offers the advantage of readily adapting the parameters that take into account the influence of genetic determinants such as the p53 gene status. Information about numerous genetic determinants for various tumors is ever-accumulating (e.g. through the use of microarrays). Once reliable results are available and the prognostic value of specific genetic data becomes well established, the status of the relevant genetic indicators can be easily incorporated into the simulation model, leading to its substantial clinical refinement.

Obviously, experimental and clinical feedback should always be used in order to improve the reliability of the model. The software system is undergoing a clinical adaptation procedure, by comparing the model “predictions” with clinical data before, during and after a radiotherapy course. In parallel, all the involved phenomena are constantly being studied, in order to keep pace with the ever-accumulating scientific knowledge. The model’s discrete and modular character facilitates significantly this procedure.

A simulation model of tumor response to other treatment modalities, such as chemotherapy has already been presented by our group (Stamatakos et al. 2005). The chemotherapy model presented in this article provides a theoretical support for the observed superiority of the standard administration schedule of the Temozolomide agent commonly used to treat glioblastoma multiforme. A good qualitative agreement of the model’s predictions with clinical experience supports the applicability of the presented approach to chemotherapy optimization. Furthermore, simulation of the response of the adjacent normal tissues to irradiation and other treatment schemes is under development.

It should be stressed that a statistical-probabilistic component will always be an innate characteristic of any oncological simulation model, as cancer itself is a highly complex and partly unpredictable disease. Nevertheless, the use of advanced computer simulation models is a valuable tool to study the involved biological phenomena in an objective and functional way.
